# Decrease in wearable-based nocturnal sleep efficiency precedes epileptic seizures

**DOI:** 10.3389/fneur.2022.1089094

**Published:** 2023-01-11

**Authors:** Laura Gagliano, Tian Yue Ding, Denahin H. Toffa, Laurence Beauregard, Manon Robert, Frédéric Lesage, Mohamad Sawan, Dang K. Nguyen, Elie Bou Assi

**Affiliations:** ^1^Institute of Biomedical Engineering and the Department of Electrical Engineering, Polytechnique Montréal, Montreal, QC, Canada; ^2^Centre de Recherche du Centre Hospitalier de L'Université de Montréal (CRCHUM), Montreal, QC, Canada; ^3^CenBRAIN, Westlake University, Hangzhou, China; ^4^Department of Neuroscience, Université de Montréal, Montreal, QC, Canada

**Keywords:** epilepsy, Hexoskin, seizure risk, seizure forecasting, sleep quality (SQ), wearable

## Abstract

**Introduction:**

While it is known that poor sleep is a seizure precipitant, this association remains poorly quantified. This study investigated whether seizures are preceded by significant changes in sleep efficiency as measured by a wearable equipped with an electrocardiogram, respiratory bands, and an accelerometer.

**Methods:**

Nocturnal recordings from 47 people with epilepsy hospitalized at our epilepsy monitoring unit were analyzed (304 nights). Sleep metrics during nights followed by epileptic seizures (24 h post-awakening) were compared to those of nights which were not.

**Results:**

Lower sleep efficiency (percentage of sleep during the night) was found in the nights preceding seizure days (*p* < 0.05). Each standard deviation decrease in sleep efficiency and increase in wake after sleep onset was respectively associated with a 1.25-fold (95 % CI: 1.05 to 1.42, *p* < 0.05) and 1.49-fold (95 % CI: 1.17 to 1.92, *p* < 0.01) increased odds of seizure occurrence the following day. Furthermore, nocturnal seizures were associated with significantly lower sleep efficiency and higher wake after sleep onset (*p* < 0.05), as well as increased odds of seizure occurrence following wake (OR: 5.86, 95 % CI: 2.99 to 11.77, *p* < 0.001).

**Discussion:**

Findings indicate lower sleep efficiency during nights preceding seizures, suggesting that wearable sensors could be promising tools for sleep-based seizure-day forecasting in people with epilepsy.

## 1. Introduction

Epilepsy is a chronic neurological condition which affects about 60 million people worldwide and is characterized by a predisposition to spontaneous and recurrent epileptic seizures (1). It has long been known that sleep plays an important role in epilepsy with evidence of a bidirectional relationship between them (2). On one hand, sleep loss is commonly reported by patients as a seizure precipitant (3). On the other hand, epileptic seizures can disrupt sleep the next night, decreasing the time spent in rapid eye movement (REM) sleep, increasing non-REM stage 1 sleep, and decreasing sleep efficiency (4).

Monitoring sleep could possibly improve the management of seizures for people with epilepsy (PWE) (3, 5, 6). For example, it has been shown that the recognition and treatment of obstructive sleep apnea can improve seizure control (7). One might also even envision forecasting seizures based on sleep monitoring. While sleep deprivation is frequently used in epilepsy monitoring units to provoke seizures for clinical observation, sleep quality has seldom been leveraged to forecast seizure risk (5). One recently published study using intracranial electroencephalography (EEG) reported better-than-chance seizure forecasting performances in 5 of 8 patients using sleep data obtained with an automatic classifier of sleep stages (8). Another study conducted on long-term recordings of 10 patients from the same dataset found that overall, an increase of 1.66 h or more in total sleep duration was correlated with a significant reduction in seizure risk of 27% in the following 48 h (9). While both studies present promising results regarding the utility of poor sleep as an indicator of higher seizure likelihood, they were conducted on a relatively small and selective cohort of patients and forecasts were made hourly. On the contrary, Proix et al. demonstrated the possibility of forecasting daily seizure risk up to 3 days prior to seizure occurrence based on interictal epileptiform activity in long-term intracranial EEG (10). This study has inspired new avenues of research, notably the use of long horizons (24-h periods) for forecasting daily seizure risk. Obviously, long-term continuous monitoring of sleep at home using intracranial EEG is impractical due to the complexity of the set-up required to acquire the signal.

Traditional non-invasive sleep analysis is based on polysomnography (PSG) which combines scalp-EEG, electro-oculography, electromyography, and respiration recordings for the detailed analysis of sleep macrostructure. PSG is currently the most accurate sleep staging technique and the gold standard for short-term sleep-disorder diagnosis in dedicated sleep clinics (11). However, the sensors and analysis involved are impractical for long-term sleep monitoring. Wearable devices could provide a novel solution for measuring sleep in this respect. For example, actigraphy, the recording of body activity through a wearable accelerometer, has been accepted and extensively used to measure sleep quality in both sleep medicine and research (12–14). More recently, automatic sleep quality analysis based on electrocardiography (ECG), respiration, and movement has been demonstrated using a wearable smart shirt (Hexoskin by *Carré Technologies Inc*.). The Hexoskin sleep analysis algorithm, which classifies signals into wake and sleep, was developed and validated on healthy young adults (15). This wearable leverages novel integrated sensors to combine multimodal signals which allows it to recognize wake and sleep with high accuracy. While several commercially available wearable devices have been leveraged for sleep analysis, such approaches have seldom been explored in PWE.

In this study, we used the Hexoskin smart shirt to monitor nocturnal sleep in a large cohort of PWE in a controlled clinical setting. We aimed to assess the feasibility of monitoring sleep quality in PWE using a novel smart shirt and to evaluate whether wearable-measured changes in nocturnal sleep quality are associated with the occurrence of seizures within the 24 h following wake.

## 2. Materials and methods

### 2.1. Patient recruitment

Patients admitted to the University of Montreal Hospital Center Epilepsy Monitoring Unit (EMU) (April 2019 to March 2021) were recruited on the day of their admission to wear the smart shirt during their hospitalization. For most patients, the Hexoskin smart shirt was installed at the beginning of their hospitalization and removed prior to their discharge by research personnel (median starting day: day 2 following EMU admission). On average, patients wore the Hexoskin smart shirt for 9.2 days [media*n* = 9 days, range = (2, 24)] out of 11.8 days at the EMU [media*n* = 11, range = (3, 25)]. The shirts were removed and replaced with clean ones when patients took a shower. Patients underwent simultaneous video-EEG monitoring with the Nihon Kohden system which allowed for all seizures to be accurately annotated by neurologists. A total of 393 days of continuous smart-shirt data from 53 patients, with a confirmed diagnosis of epilepsy, were recorded. Six patients who had seizures every day/night were excluded to avoid reporting overly optimistic results. Recordings from 47 patients [19 females, mean ± s.d. age: 36.32 ± 13.37 years, range = (19, 66)] were analyzed. Both patients with focal (43/47) and generalized (4/47) epilepsy were recruited and included in the study. For patients with focal epilepsy, the suspected localization of the epileptic focus is provided in the Supplementary Table 1. This study was approved by our Institutional Ethics Research Board and performed in accordance with its guidelines and regulations (18.091). All patients signed an ethical board-approved written informed consent prior to participation.

### 2.2. Signal acquisition

#### 2.2.1. Multimodal recordings

**Figure 1:**
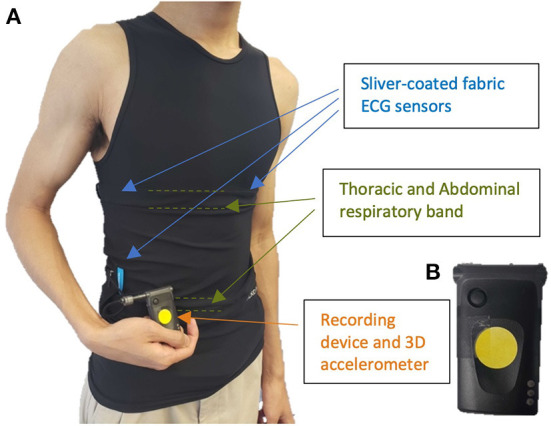
**(A)** smart biosensor shirt equipped with integrated electrocardiogram (ECG), respiratory bands, and **(B)** detachable recording device with incorporated 3D accelerometer.

**Figure 2:**
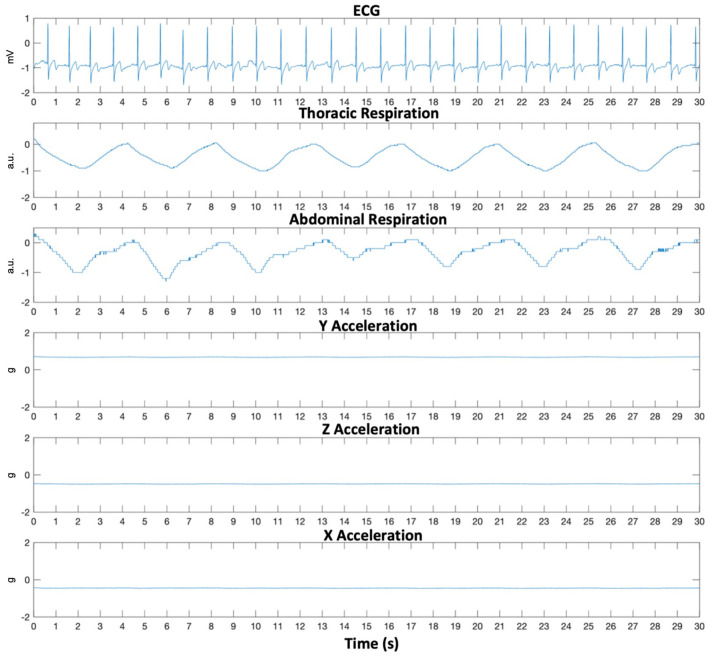
Thirty-second raw multimodal smart shirt recording during nocturnal sleep. ECG, Electrocardiogram sampled at 256 Hz; Respiration sampled at 128 Hz each in arbitrary units (a.u.); Acceleration sampled at 64 Hz each.

Patients wore the Hexoskin smart shirt (*Carré Technologies Inc*.), a garment embedded with a single-lead ECG (256 Hz) and two (one thoracic and one abdominal) inductive respiratory bands (128 Hz each). It also includes a detachable telemetry device which holds a three-axis accelerometer (64 Hz) and collects, preprocesses, and transmits all recordings to the Hexoskin Connected Health Platform. The smart shirt sensors as well as an example of raw acquired recordings are shown in Figures 1, 2, respectively. Patients were fitted with either the women's or men's shirt following the Hexoskin sizing guide. While the shirts were changed every few days when patients showered, the detachable telemetry devices, containing the battery, were replaced every 24 h by research personnel.

### 2.3. Data selection and classification

#### 2.3.1. Data annotation

**Figure 3:**
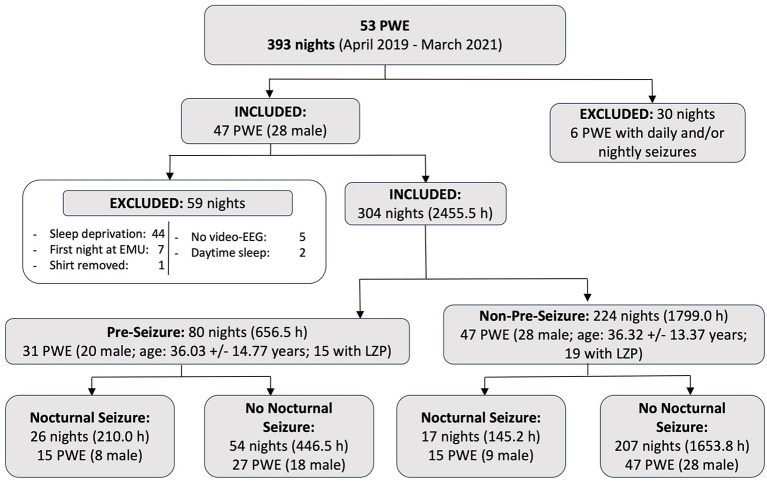
Summary of data collected and included based on inclusion and exclusion criteria. A total of 393 nights of continuous recordings were acquired from 53 patients who were recruited at our Epilepsy Monitoring Unit between April 2019 and March 2021. Of these, 304 nights (2,455.5 h) were included and analyzed in this study. PWE, people with epilepsy; LZP, patients who took lorazepam at least once during their hospitalization; Nocturnal Seizure, nights with at least one clinical nocturnal seizure during sleep; No Nocturnal Seizure, nights with no clinical seizure during sleep.

Seizures were annotated by epileptologists based on video-EEG recordings (blinded to the Hexoskin data). Each night was then labeled as either “pre-seizure” if it was followed by at least one epileptic seizure in the 24-h post-awakening (including nocturnal seizures occurring during the following night) or “non-pre-seizure” if it was not followed by any epileptic seizure in the 24-h post-awakening. The presence or absence of nocturnal seizures was also noted as a dichotomous variable. A detailed description of the data inclusion criteria is provided in Figure 3.

#### 2.3.2. Nocturnal data selection and segmentation

For all patients, in accordance with standard sleep analysis common practices, the first night at the EMU was excluded to avoid contaminating the data with recordings during the adaptation period. The first night of sleep analysis is often accompanied by lower sleep efficiency due to a combination of factors such as a change in environment and stress (16). Moreover, nights during which there was a full-night sleep deprivation (to trigger seizures for clinical purposes) or significant sleep during the preceding day (a single period of continuous sleep lasting at least 4 h) were also excluded from further analysis as to not bias sleep data with recordings which do not represent true physiological sleep patterns. All other nights from the beginning, middle, or end of the patients' hospitalization were included regardless of medication changes. An important advantage of the EMU is that it is a controlled environment for various sleep-related factors across patients (e.g., lighting, sleep schedule, diet, temperature, alcohol consumption, comfort, physical activity level, etc.). In addition, patients are monitored 24 h per day with high-density video which was recorded and saved. The lights off and lights on times were determined visually by analyzing video recordings. Lights off was labeled as the moment when the patient expressed their intention to sleep (e.g., when a patient puts away their phone or their book and lies down immobilized on their bed) and lights on as the moment when the patient woke up for the last time and no longer expressed any desire in sleeping (e.g., got out of bed, turned on the lights, inclined their bed). The Hexoskin sleep analysis algorithm was then run on continuous recordings between lights on and off to extract metrics of sleep quality.

### 2.4. Sleep metric extraction

The Hexoskin sleep algorithm used was previously developed and validated in young healthy adults. The algorithm analyzes multimodal recordings to classify continuous, non-overlapping 20-second segments as either sleep or wake with 90.8% agreement with standard PSG recordings (15). The Hexoskin Connected Health Platform then calculates descriptive sleep metrics based on this classification: time in sleep (minutes), time in wake (minutes), sleep latency (delay between lights off and sleep onset), and sleep efficiency (total percentage of sleep between sleep onset and offset). From the three Hexoskin metrics for each night, the following two commonly used sleep metrics, time of waking after sleep onset (WASO) in minutes and the total sleep duration (hours), were calculated:


(1) $$\begin{array}{l} \text{WASO }=\text{ time in Wake}-\text{sleep latency} \end{array}$$

(2) $$\begin{array}{l} \text{Total sleep duration }=\text{ time in sleep }+\text{time in WASO} \end{array}$$

The four sleep metrics which were analyzed in this study are the following: total sleep duration (hours), sleep latency (minutes), WASO (minutes), and sleep efficiency (%).

### 2.5. Statistical analysis

The four sleep metrics were statistically compared between pre-seizure nocturnal sleep and non-pre-seizure nocturnal sleep to evaluate whether seizure days (24 h) are preceded by significantly lower quality of sleep: decreases in sleep efficiency or total sleep duration and increases in sleep latency or WASO. One tailed Welch *t-*tests or Wilcoxon rank-sum tests were then used according to the distribution of the data. Bonferroni correction was applied to account for multiple comparisons with an adjusted alpha level of 0.0125 (17). Logistic regression analysis was then conducted for sleep efficiency and WASO to evaluate the association between reduced sleep quality and the odds of seizure occurrence the following day.

#### 2.5.1. Pre-seizure vs. non-pre-seizure nocturnal sleep

Three sub-analyses were conducted between pre-seizure and non-pre-seizure nights. The first included all nights (304 nights, 2,455.5 h), corresponding to 80 pre-seizure nights and 224 non-pre-seizure nights. This analysis was conducted to evaluate the existence of an overall significant difference in sleep metrics during nocturnal sleep in the eve of a clinical seizure. To assess the possible effect of nocturnal seizures during sleep, the second analysis compared sleep metrics between pre-seizure nights and non-pre-seizure nights including only nights without a nocturnal seizure (261 nights), corresponding to 54 pre-seizure nights and 207 non-pre-seizure nights. Similarly, the third analysis included only nights with the presence of at least one nocturnal seizure (43 nights), corresponding to 26 pre-seizure nights and 17 non-pre-seizure nights.

#### 2.5.2. Effect of nocturnal seizures on sleep metrics

Due to the direct link between the occurrence of clinical seizures during sleep and sleep quality, a sub-analysis to study the effect of nocturnal seizures on sleep metrics was also performed. The four sleep metrics of all nights with at least one nocturnal seizure (43) were compared to nights without a nocturnal seizure (261) regardless of whether the night was pre-seizure or non-pre-seizure. The relationship between the presence of nocturnal seizures and the night being a pre-seizure night was also analyzed using a Chi-Square test of independence.

#### 2.5.3. Potential effect of anti-seizure medication on sleep efficiency

In the EMU, patients' anti-seizure medications are often reduced or stopped. There were no medication changes prior to EMU admission. Reduction in anti-seizure medication is known to directly (i.e., modulate sleep efficiency) (18, 19) and indirectly (i.e., gradually increase seizure risk which in turn reduces sleep efficiency) (4) affect sleep. Lorazepam, a benzodiazepine sometimes prescribed to PWE in the EMU as a rescue medication with a relatively short half-life, increases sleep efficiency and reduces seizure risk in a relatively short amount of time as compared to other anti-seizure medications which have a longer half-life (20). Lorazepam is the only rescue medication which was prescribed to patients in the cohort either to prevent a seizure during nuclear imaging or to treat a seizure cluster. To analyze its potential direct confounding effect on sleep, the sleep efficiency of nights of patients who had taken lorazepam during their admission (according to their medical chart) were compared to that of patients who did not take lorazepam using the Wilcoxon rank-sum test.

## 3. Results

### 3.1. Classification of nocturnal sleep

Of the 53 patients recruited, 47 were included for analysis. During their hospitalization, 13 patients solely had focal to bilateral tonic-clonic seizures, 12 only had focal impaired awareness seizures, 11 only had focal aware seizures, 7 experienced mixed types of focal seizures, and 4 had generalized tonic-clonic seizures. Prior to analysis, 59 nights were excluded due to sleep deprivation, the first night at the EMU, significant daytime sleep (>4 h of continuous sleep), missing video-EEG recordings, and the smart shirt removed by the patient. A total of 304 nights (2,455.5 h) were included, according to the criteria depicted in Figure 3. There were 80 pre-seizure nights [26 included one or more nocturnal seizure(s)] and 224 non-pre-seizure nights [17 included one or more nocturnal seizure(s)].

### 3.2. Lower sleep efficiency during pre-seizure nocturnal sleep

#### 3.2.1. Overall analysis of pre-seizure and non-pre-seizure nights

**Figure 4:**
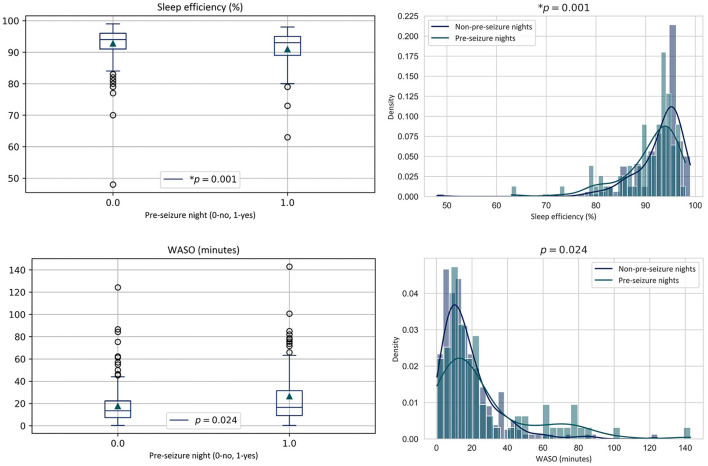
Sleep metric distributions of all nocturnal recordings followed by at least one clinical seizure occurring within the 24 h following wake (Pre-seizure) vs. those where no seizure occurred within the 24 h following wake (Non-pre-seizure). WASO, time in wake after sleep onset.

First, the four sleep metrics of all pre-seizure nights (*n* = 80) were statistically compared to those of all of non-pre-seizure nights (*n* = 224). Total sleep duration was analyzed with the Welch *t*-test. The other variables were analyzed with the Wilcoxon rank-sum test. Significantly lower sleep efficiency was observed in pre-seizure nights compared to non-pre-seizure nights (μ = 90.92 % < 92.75 %; *p* = 0.001) at a Bonferroni-corrected alpha of 0.0125. Increased time in WASO during pre-seizure nights (μ = 26.48 min > 17.67 min; *p* = 0.024) was significant at 95% confidence only before correcting for multiple comparisons. Distributions of these sleep metrics are shown in Figure 4. Interestingly, while decreased sleep efficiency caused in part by increased WASO in pre-seizure nights was expected, the distribution of the WASO suggests that two clusters of pre-seizure nights exist: nights with WASO (0–40 min) and nights with high WASO (>40 min). This is quantified by the high standard deviation for the pre-seizure nights (27.38 min) compared to the non-pre-seizure group (15.97 min) shown in Table 1. A possible explanation for these two clusters of nights is the occurrence of one or more clinical nocturnal seizure(s) during sleep which causes the patient to wake up, hence increasing their WASO relative to nights without any nocturnal seizure (mean WASO = 36.94 > 21.45 min, respectively), and consequently decreasing both the percentage of time in sleep and their sleep efficiency during that night. In accordance with these findings, the Chi-Square test showed that significantly more pre-seizure nights had nocturnal seizures than non-pre-seizure nights (*p* < 0.001).

**Table 1:** Statistical analysis results.

**Parameter**	**Pre-seizure vs. non-pre-seizure**									**With vs. without nocturnal seizures**		
	**All 304 nights** **(mean ±s.d.)**			**43 nights with nocturnal seizure(s)** **(mean ±s.d.)**			**261 nights without nocturnal seizure** **(mean ±s.d.)**			**All 304 nights** **(mean ±s.d.)**		
	**Pre- seizure** ***n =* 80**	**Non-pre-** **seizure** ***n =* 224**	***p*** **value**	**Pre- seizure** ***n* = 26**	**Non-pre- seizure** ***n* = 17**	***p* value**	**Pre- seizure** ***n* = 54**	**Non-pre-** **seizure** ***n* = 207**	***p* value**	**Nocturnal** **seizure** ***n* = 43**	**No** **nocturnal** **seizure** ***n* = 261**	***p* value**
Sleep latency (min)	17.81 ± 13.34	18.06 ± 22.19	0.045	18.74 ± 16.02	12.31 ± 14.5	0.159	17.36 ± 11.98	18.53 ± 22.66	0.045	16.2 ± 15.59	18.29 ± 20.89	0.815
Total sleep duration (h)	7.91 ± 2.062	7.73 ± 1.99	0.753	7.77 ± 2.04	8.34 ± 2.74	0.219	7.98 ± 2.09	7.68 ± 1.91	0.841	7.99 ± 2.32	7.74 ± 1.95	0.875
Sleep efficiency (%)	90.92 ± 6.108	92.75 ± 5.48	0.00139^*^	88.88 ± 6.09	88.65 ± 11.71	0.213	91.91 ± 5.93	93.09 ± 4.51	0.033	88.79 ± 8.62	92.84 ± 4.85	1.712e-04^*^
Time WASO (min)	26.48 ± 27.38	17.67 ± 15.97	0.024	36.94 ± 34.44	35.69 ± 31.27	0.549	21.45 ± 21.88	16.19 ± 13.09	0.138	36.44 ± 32.85	17.28 ± 15.42	2.739e-05^*^

* Denotes significance after Bonferroni correction *p* < 0.0125. WASO, Wake After Sleep Onset; s.d., standard deviation.

#### 3.2.2. Separation of nights with and without nocturnal seizures

Pre-seizure and non-pre-seizure sleep metrics were then compared during nights with and without one or more nocturnal clinical seizure separately to evaluate if the presence of nocturnal seizures during sleep was a confounding factor. No significant difference in sleep metrics between pre-seizure and non-pre-seizure nights was found. However, before correcting for multiple comparisons, sleep efficiency was significantly lower for pre-seizure nights than non-pre-seizure nights without a nocturnal seizure at 95% confidence (μ = 93.09 > 91.91%; *p* = 0.033). The reduced sample sizes resulted in low statistical power for these comparisons which may have caused any existing differences in other sleep metrics to not be captured. Average values of the metrics per group as well as *p* values are detailed in Table 1.

### 3.3. Nocturnal seizures are associated with increased WASO and decreased sleep efficiency

Since the occurrence of nocturnal seizures was significantly greater in pre-seizure nights according to the Chi-Square test described in Section 3.2.1, the effect of nocturnal seizures on sleep metrics was assessed in a *post-hoc* analysis. The four sleep metrics for all the nights with at least one nocturnal seizure (*n* = 43) were compared to all of the nights without a nocturnal seizure (*n* = 261), regardless of whether or not the nights were pre-seizure or non-pre-seizure, using the Wilcoxon rank-sum test. Significantly lower sleep efficiency was observed in nights with nocturnal seizures than nights without (μ = 88.79 < 92.84%; *p* < 0.001). Nights with nocturnal seizures also showed significantly greater WASO (μ = 36.44 > 17.28 min; *p* < 0.001).

**Figure 5:**
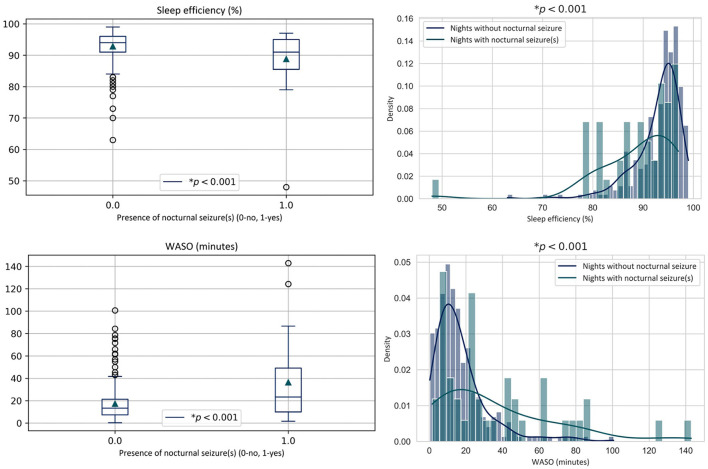
Sleep metric distributions of all recordings containing at least one clinical nocturnal seizure vs. those with no nocturnal seizure. WASO, time in wake after sleep onset.

Distributions of significant metrics, illustrated in Figure 5, show that nights with nocturnal seizures show more dispersed values, which is also reflected in higher standard deviations in Table 1. More specifically, sleep efficiency and WASO appear to show clusters in the probability distribution which were also observed in the global analysis of pre-seizure and non-pre-seizure sleep.

### 3.4. Lower sleep quality is associated with higher odds of seizure occurrence the following day

Each standard deviation decrease in sleep efficiency (5.70%) was associated with a 1.25-fold increase in the odds of seizure occurrence in the 24 h following wake according to a logistic regression analysis (95% CI: 1.05 to 1.42, *p* = 0.02). Each standard deviation increase in time in WASO (19.96 min) was associated with a 1.49-fold increase in the odds of seizure occurrence in the 24 h following wake (95% CI: 1.17 to 1.92, *p* < 0.01). Given the high occurrence of nocturnal seizures during pre-seizure nights, the odds of seizures occurring after a night disrupted by one or more nocturnal seizures were also assessed. Interestingly, there was a 5.86-fold increased odds of seizure occurrence in the 24 h following wake associated with a nocturnal seizure occurring during sleep (OR 5.86, 95% CI: 2.99 to 11.77, *p* < 0.001).

### 3.5. Potential confounding effect of anti-seizure medication on sleep efficiency

Nineteen patients (40.42%) received lorazepam at least once during their hospitalization for various reasons (e.g., prior to being transported to the nuclear medicine department or because of a seizure cluster). The 151 nights of patients who took lorazepam had on average an increased sleep efficiency of 0.73% as compared to the 153 nights of the 27 patients who did not (μ = 92.64 > 91.91%). This difference was not statistically significant according to the Wilcoxon rank-sum test (*p* = 0.24). In addition, a thorough investigation of the reduction/discontinuation of other anti-seizure medication was conducted. For each patient, specific medications and dose changes are provided in Supplementary Table 1. Unfortunately, given the heterogeneity of medication types and dosage changes, patients could not be grouped in a representative way that would allow for a statistical analysis of the indirect effect of the addition or reduction of anti-seizure medication on sleep efficiency because over 40 unique medication dosage combinations were present. Seizure clusters are also reported in Supplementary Table 1. Only 4/47 patients had seizure clusters.

## 4. Discussion

In this study, metrics characterizing nocturnal sleep quality were collected using a novel smart shirt from a cohort of 47 PWE (2,455.5 h) in the EMU. We evaluated the existence of lower sleep quality during nights followed by a seizure in the 24 h post-awakening. To our knowledge, this is the first study to perform sleep monitoring using a multimodal smart shirt in PWE to quantitatively evaluate changes in sleep quality preceding seizure occurrence in a clinical setting.

Overall, significantly lower sleep efficiency was observed in nights followed by at least one clinical seizure compared to nights not followed by seizures. These findings show promise for the possibility of forecasting seizure risk based on wearable sleep monitoring and are concordant with recent investigations on early seizure forecasting based on interictal epileptiform activity (10), patient-reported prodromes (21, 22), and patient-specific seizure and heart rate cycles (23, 24). Combining wearable sleep monitoring with other non-invasive modalities could potentially improve current forecasting performances and facilitate the collection of long-term multimodal databases (5, 6). This study will guide our future investigations which aim at developing a probabilistic seizure-day forecasting algorithm based on wearable sensors. It was recently shown, in long-term intracranial EEG recordings from 10 patients, that an increase of 1.66 ± 0.52 h of total daily sleep reduced the odds of seizure occurrence in the following 48 h by 27% (9). Although our results show no significant differences in the sleep period length, the sleep efficiency calculated by the Hexoskin algorithm is based on the percentage of time asleep during the night (15). While increased WASO during pre-seizure nights was only significant before Bonferroni correction, logistic regression models showed that it was significantly associated with increased odds of seizure occurrence the following day. It should be noted that we have chosen to adopt a conservative approach for multiple comparisons correction in the interpretation of our results to avoid an inflated Type I error rate, while also reporting actual *p* values (17). Furthermore, the clusters observed in the pre-seizure night WASO distribution suggest that wake caused by nocturnal seizures may be an early precursor of higher seizure likelihood the following day. A larger number of nights with nocturnal seizures is necessary to statistically quantify this possible correlation. Moreover, the odds of having one or more seizures in the 24 h after waking was increased 5.86-fold after nights with nocturnal seizures as compared with nights without. The impact of nocturnal seizures on the sleep quality of the night during which they occur was also studied. The decrease in sleep efficiency in nights with nocturnal seizures, as measured by the Hexoskin smart shirt, is in accordance with previous findings (4, 9). The increase in WASO in seizure nights is also consistent with the literature which shows that awakenings and arousals often follow nocturnal seizures (9, 25).

While sleep efficiency was significantly lower during nights preceding seizures and was associated with greater odds of seizure occurrence the following day, further studies investigating the mechanisms underlying the relationship between sleep efficiency and seizure occurrence are required to evaluate the causality between them. This metric was calculated based on the classification of periods of sleep and wake and did not take into account patient-specific factors which may independently provoke seizure occurrence such as withdrawal of anti-seizure medication, variations in stress levels, and baseline seizure frequency. The findings of this study suggest that disrupted sleep (measured as low sleep efficiency) may be driving seizure occurrence. However, low sleep efficiency in the EMU is one of many seizure-provoking factors.

In addition, albeit being statistically significant, the differences measured in sleep efficiency and WASO between pre-seizure and non-pre-seizure nights were relatively small. We surmise that these differences could be larger at home where sleep is not as ‘controlled' as it is in the EMU. In the near future, we believe that such metrics could be combined with other personalized seizure risk markers (for example multi day cycles of seizures and/or daily levels of stress) to enhance seizure day forecasting performances. In this study, simple metrics of sleep quality measured solely by a wearable were studied. Although these metrics were based on sleep-wake classifications previously validated with polysomnography (15), more complex EEG-based metrics of sleep macro- and microstructure should be evaluated while controlling for other seizure-provoking factors. Such sleep analyses, based on multi-stage classification of PSG recordings, could potentially provide additional insight into the relationship between sleep patterns and seizure risk. Future studies exploring other quantitative sleep analysis techniques could significantly improve seizure-day forecasting capabilities (5, 6). The advantage of using a wearable device is that it allows recording multiple nights from a large cohort of patients and can be used to monitor sleep efficiency in a residential setting. Our group's future work will explore various wearable sleep monitoring techniques for seizure forecasting in an outpatient setting. Furthermore, while patients with focal and generalized epilepsies were included in the study, the majority had only focal epilepsy (43/47) of varying severity. The recordings of the patients were thus analyzed in a general manner; however, future studies involving a seizure day forecasting algorithm should report results for different seizure and epilepsy types.

Presented results provide new insights regarding the complex relationship between sleep and seizure occurrence; nonetheless, several avenues of improvement exist. In the study by Dell et al. (9), 10 patients took an average of 0.21 to 1.34 naps per day. Daytime naps are known to alter daytime sleepiness and nocturnal sleep macrostructure (26, 27); therefore, nights preceded by significant daytime sleep were excluded in this study. While considering daytime naps could potentially give more accurate sleep metrics, continuous 24-h sleep monitoring with wearable sensors goes beyond the scope of this study and would require extensive reviewing of continuous HD video for potential naps to be labeled. Furthermore, the nature of the activities done by patients who are hospitalized at the EMU would render daytime naps undistinguishable since patients are not engaging in ordinary real-life activities (e.g., cooking, working, and exercising) and their sleep schedule is relatively regular. Despite that total 24-h sleep was not analyzed in this study, the significantly lower nocturnal sleep efficiency measured using the smart shirt is promising for future studies in a real-life setting where other sleep parameters may be evaluated. In this study, the inclusion of a large number of patients and recording days was prioritized to gain a global understanding of smart shirt-based sleep quality in PWE. However, comparing eventual forecasting performances based on nocturnal and 24-h sleep would be of great importance in future work. We also recognized that EMU patients undergo several medication changes which could have directly or indirectly affected sleep metrics. In our study, only the effect of lorazepam was assessed since it is the only fast-acting anti-seizure medication known to increase sleep which was administered to included patients (19). Changes in anti-seizure medication for each patient are provided as Supplementary Table 1 to assist future analysis. Since no significant effect of lorazepam on sleep efficiency was observed, patients who took the medication during their hospitalization were included in the study. This study was conducted in the EMU providing two important advantages: (1) the EMU allows control of various sleep-related factors across patients (e.g., lighting, schedule, diet, temperature, alcohol consumption, comfort, physical activity level, etc.); and (2) simultaneous video-EEG allows for accurate diurnal and nocturnal seizure annotation. Future studies on sleep-based seizure forecasting algorithms developed for at-home settings could benefit from patient-specific baseline sleep quality, seizure frequency, and epileptogenic zone localization. In addition, patient-specific factors such as anti-seizure medication dosages, age, and sex which have been known to significantly affect sleep structure, should be considered when forecasting seizure likelihood (28, 29). Thus, while our results suggest that metrics of sleep quality measured using a wearable show promise as indicators of higher seizure susceptibility following wake, we must be cautious to infer causality between poor sleep quality and seizure occurrence until more studies are performed.

## 5. Conclusion

Our findings reveal lower sleep efficiency, measured by the Hexoskin smart shirt, during nights preceding seizures in a large cohort of patients with epilepsy. Increased wake during nocturnal sleep and the occurrence of seizures during nocturnal sleep could be early precursors of increased seizure odds the following day. These findings will guide future work on the development of probabilistic seizure-day forecasting based on long-term wearable sleep monitoring. Further studies looking at different seizure types, patient-specific baseline sleep macrostructure, and the effect of age and sex in a real-life setting would greatly improve our understanding of the bidirectional relationship between sleep and seizures.

## Data availability statement

The datasets presented in this article are not readily available because sharing of raw data is conditional to approval by our Institution's Research Ethics Board. Requests to access the datasets should be directed to EB, elie.bou.assi.chum@ssss.gouv.qc.ca.

## Ethics statement

This study involving human participants was reviewed and approved by the Research Ethics Board of the Centre de Recherche du Centre Hospitalier de L'Université de Montréal. The patients/participants provided their written informed consent to participate in this study.

## Author contributions

DN and EB are equally contributing principal investigators. LG and TD wrote the main manuscript text and performed the statistical analysis. LG, TD, DT, LB, MR, and EB performed the study design and data retrieval. TD, LG, and EB prepared the figures. All authors contributed to the article and approved the submitted version.
