# Supplementary material for: Decrease in wearable-based nocturnal sleep efficiency precedes epileptic seizures
Source: Front Neurol. 2023 Jan 11;13:1089094. doi: 10.3389/fneur.2022.1089094 (PMC9875007; doi:10.3389/fneur.2022.1089094)
Supplement: Supplementary file 1 [file Data_Sheet_1.PDF]

Supplementary Table 1: Summary of patient admission days, anti-seizure medications (ASMs), and lorazepam (LZP)

Patient ID	Sex	Age (yrs)	Epilepsy type*	Main seizure type	EMU Days	Study Days	ASMs at admission	ASM addition (day)	Lowest ASM dose (day)	LZP dose (day)	LZP reason
001	M	47	Bitemporal	FBTCS	17	7-17	PHT 200 BID<br>LTG 150 BID		0 (7)<br>0 (7)	3mg (9)<br>2mg (11)<br>1mg (12)<br>2mg (14)<br>2mg (15)	cluster<br>cluster<br>cluster<br>psychosis<br>cluster
002	F	32	Right temporal	FBTCS	25	2-25	LCM 200 BID<br>ESL 600		0 (8)<br>0 (14)	1mg (3)<br>1mg (12)<br>1mg (14)<br>1mg (22)	sustained<br>SPECT<br>other*<br>other*
003 (first stay)	M	29	Right temporal	FAS	23	2-23	BRV 50 BID<br>LTG 200 BID<br>CBZ CR 800 BID		0 (3)<br>0 (14)<br>0-400 (15)		
003 (second stay)	M	31	Right temporal	FAS	9	7-9	BRV 100 BID<br>LCM 200 BID<br>LTG 200 BID		0-25 (4)<br>0 (3)<br>200 BID (1)	1mg (5)	SPECT
004	M	49	Right temporal	FBTCS	18	3-6	CBZ CR 400-600<br>LCM 200 BID		100 BID (5)<br>0 (5)	1mg (6)<br>1mg (15)	sustained<br>SPECT
005	F	59	Left temporal	FBTCS	14	6-14	VPA 500-250-500<br>CLB 5-10<br>PER 2 HS		0 (7)<br>0-2.5 (9)<br>0 (7)	1mg (11)	sustained
006	M	38	Generalized	GTCS	12	3-9	PHT 100 HS<br>LEV 1500 BID<br>VPA 750 BID	TPM 100 BID (2)<br>BRV 100 BID (4)<br>LTG 25 BID (10)	0 (2)<br>0 (4)<br>0 (7)<br>0 (9)<br>100 BID (4)<br>100 BID (10)	2mg (1)<br>1mg (7)	sustained<br>sustained
007	M	43	Right frontotemporal	FIAS	14	3-14	LCM 150-250<br>CBZ CR 800 BID	PER 2 HS (11)	0 (7)<br>200-400 (10)<br>2 HS (11)		
008	M	58	Left frontotemporal	FBTCS	15	3-13	LEV 1250 BID<br>LTG 100 BID<br>PHT 100 BID<br>CNZ 0.5 HS		250 BID (8)<br>0 (8)<br>0 (3)<br>0.5 HS (1)		
009	M	55	Right temporal	FIAS	19	4-18	BRV 100 BID		0 (3)		

							CBZ 400-600<br>CLB 30 HS<br>Pb 30-60	PGB 150 BID (17)	0 (11)<br>0 (10)<br>0 (4)<br>150 BID (17)		
010	M	22	Generalized	GTCS	5	2-5	VPA 125 BID<br>LCM 250 BID<br>CLB 10 BID		125 BID (1)<br>250 BID (1)<br>0 (3)	1mg (4)	SPECT
011	F	35	Right temporoparietal	FAS	3	2-3	LCM 200 BID		200 BID (1)		
012	M	46	Left temporal	FBTCS	17	5-17	CBZ CR 600 BID<br>CLB 30 HS<br>LTG 200 BID		100 BID (6)<br>0 (7)<br>0 (6)	2mg (8)<br>2mg (12)	sustained<br>sustained
013	F	27	Bitemporal	FAS, FIAS	24	2-14	LCM 100-150<br>LEV 1750 BID<br>LTG 25 BID		0 (4)<br>0 (15)<br>0 (12)		
014	M	28	Left temporoinsular	FBTCS	5	2-5	BRV 100 BID<br>LCM 200 BID		50 BID (2)<br>100 BID (2)		
015	M	48	Right temporal	FBTCS (controlled), PNES	8	2-7	LEV 750 BID<br>CBZ CR 400 BID<br>PGB 150 HS		750 BID (1)<br>0 (3)<br>150 HS (1)		
016	F	66	Left temporal	FAS	12	2-12	CBZ CR 300 BID<br>LCM 50 BID		0 (3)<br>50 BID (1)		
017	F	24	Combined generalized and multifocal	GTCS, FBTCS	10	2-10	VPA 500 TID<br>TPM 100 BID<br>LEV 1500 BID		0 (3)<br>0 (3)<br>0 (8)		
018	F	42	Right temporal	FBTCS	16	2-16	OXC 900-1200<br>CLB 10-20		300 BID (9)<br>10 BID (6)	3mg (11)	cluster
019	M	22	Right insular	FIAS	15	3-14	LEV 2000 BID<br>VPA 1250 BID<br>TPM 300 BID		1000 BID (12)<br>0 (12)<br>0 (5)	1mg (7)<br>2mg (8)<br>2mg (13)<br>2mg (15)	sustained<br>MRI<br>sustained<br>sustained
020	F	20	Right frontal	FBTCS	19	4-18	PHT 200-300<br>OXC 750-900		0 (6)<br>0 (8)	2mg (9)<br>2mg (11)	MRI<br>sustained
021	M	55	Bitemporal	FAS	7	5-7	CLB 5-10<br>CBZ CR 600 BID<br>TPM 100 BID	LCM 50 BID (5)	10 HS (4)<br>600-0 (2)<br>100 BID (1)<br>50 BID (5)		

022	M	34	Bitemporal	FIAS,<br>FBTCS	12	2-12	ESL 1000 BID<br>TPM 100 BID<br>CLB 30 HS		0 (3)<br>0-25 (9)<br>0-10 (5)	2mg (6)	sustained
023	F	38	Right temporal	FAS	10	3-11	LEV 1500 BID<br>LTG 125 BID		1500 BID (1)<br>125 BID (1)		
024	F	53	Left temporal	FAS	8	2-7	CBZ CR 300 BID<br>LTG 250 BID		300 BID (1)<br>0 (7)		
025	M	61	Right temporal	FIAS	12	4-12	LTG 75 BID<br>LEV 1000-500-500-500<br>CLB 10-20<br>CBZ CR 400-200-200		0 (4)<br>1000-500-500-<br>500 (1)<br>0 (6)<br>0 (5)		
026	F	33	Bitemporal	FBTCS	19	2-19	LTG 100 BID<br>VPA 375 BID		0 (8)<br>0 (8)		
027	M	22	Bilateral multifocal (right temporal +++)	FBTCS	19	7-19	ESL 800-0<br>LTG 100 BID<br>LEV 1500 BID<br>PHT 400 HS<br>CNZ 0.25-1.25		800-0 (1)<br>0 (5)<br>0 (15)<br>0 (5)<br>0 (9)	2mg (5)<br>2mg (6)	sustained<br>sustained
028	M	33	Bitemporal	FIAS	14	3-13	BRV 100 BID<br>CBZ CR 800-400-800<br>CLB 20 HS		0 (2)<br>0 (7)<br>0 (9)		
029	F	43	Bitemporal	FAS	8	2-7	CBZ CR 400-500<br>BRV 50 BID		400-500 (1)<br>50 BID (1)		
030	M	32	Right temporal	FAS	7	2-6	LCM 150 BID<br>CBZ CR 400 BID		150 BID (1)<br>400 BID (1)	0.5mg (6)	SPECT
031	M	30	Left parietal	FIAS,<br>FBTCS	8	2-8	LEV 625-750<br>TPM 25-50<br>LTG 100 BID		625-750 (1)<br>25-50 (1)<br>100 BID (1)		
032	M	19	Left temporal	FIAS	9	1-9	TPM 150 BID<br>LCM 200 BID<br>CLB 15 BID<br>CBZ CR 400 BID		0 (5)<br>200 BID (1)<br>15 BID (1)<br>400 BID (1)		
033	M	21	Left frontal	FIAS,<br>FBTCS	5	1-4	CLB 30 BID<br>LEV 1500 BID	CBZ CR 200 BID (3)	20-30 (2)<br>1000 BID (2)<br>200 BID (3)	2mg (2)	other**
034	M	31	Left temporal	FIAS,	11	1-10	PER 8 HS		0 (3)	2mg (10)	x-ray

				FBTCS			CBZ CR 400 BID<br>CLB 15 HS		0 (2)<br>0 (5)		
035	F	47	Multifocal (frontotemporal ++)	FIAS	7	2-6	LEV 1500 BID<br>CBZ CR 400-600<br>OXC 300 BID	LCM 50 BID (6)	1500 BID (1)<br>400-600 (1)<br>300 BID (1)<br>500 BID (6)		
036	F	22	Focal (unknown focus)	FIAS	11	1-11	LEV 1500-1500<br>LTG 200-200<br>CLB 5-5-10		250 BID (7)<br>0 (3)<br>0 (8)	3mg (8)	cluster (& SPECT)
037	M	19	Right frontal	FBTCS	10	1-10	CBZ CR 400 BID<br>CLB 30 BID<br>LEV 1500 BID		0 (5)<br>30 BID (1)<br>1500 BID (1)		
038	M	32	Bifrontal	FAS	11	2-10	CBZ CR 400-600<br>LCM 200 BID		0 (8)<br>0 (2)		
039	F	62	Bitemporal	FAS	6	2-6	LTG 150-100<br>CLB 10 HS	PGB 25 HS (5)	50 BID (3)<br>0 (4)<br>25 HS (5)		
040	M	21	Bitemporal	FIAS	15	2-8	CBZ CR 400 BID		0 (2)		
041	F	33	Bitemporal	FIAS	6	1-6	LTG 100 BID<br>LCM 200 BID<br>BRV 75 BID		0 (3)<br>0 (2)<br>50 BID (5)		
042	F	38	Generalized	GTCS	9	2-6	PER 4 HS<br>LTG 100 BID		0 (1)<br>0 (5)		
043	M	27	Frontal (unknown lateralization)	FIAS, FBTCS	11	1-11	LTG 200 BID<br>TPM 200 BID<br>BRV 100 BID	VPA 250 BID (7)	50 BID (4)<br>0 (5)<br>0 (3)<br>250 BID (7)		
044	M	20	Right frontal	FIAS, FBTCS	8	2-7	LCM 200 BID<br>PER 10<br>PHT 175 BID		200 BID (1)<br>7.5 (2)<br>0-100 (5)		
045	F	42	Right temporal	FIAS, PNES	6	2-6	LEV 750 BID<br>LCM 150 BID		0 (3)<br>150 BID (1)		
046	M	23	Right temporal	FIAS, FBTCS	10	2-10	BRV 50 BID<br>LCM 200 BID<br>VPA 500 BID<br>CLB 20		0 (3)<br>100-100 (4)<br>0 (3)<br>0 (4)	2mg (5)	SPECT

047	F	25	Bitemporal	FIAS, FBTCS	12	1-12	LTG 75 BID		0 (1)	1mg (4)<br>1mg (5)	sustained<br>sustained

*Epilepsy types were not validated by intracranial EEG investigations.

Abbreviations

Main seizure type
FAS: Focal aware seizure
FIAS: Focal impaired awareness seizure
FBTCS: Focal to bilateral to tonic-clonic seizure
GTCS: Generalized tonic-clonic seizure
PNES: Psychogenic nonepileptic seizure

ASM
BRV: brivaracetam
CBZ CR: carbamazepine CR
CLB: clobazam
CNZ: clonazepam
ESL: eslicarbazepine
LCM: lacosamide
LEV: levetiracetam
LTG: lamotrigine
LZP: lorazepam
OXC: oxcarbazepine
Pb: phenobarbital
PER: perampanel
PHT: phenytoin
TPM: topiramate
VPA: valproic acid

BID: administered twice per day
HS: administered before bedtime
TID: administered three times per day

Cluster: seizure cluster
Sustained: sustained seizure for several minutes
*Other: LZP administered after a prescribed sleep deprivation
**Other: LZP administered to prevent a nocturnal seizure

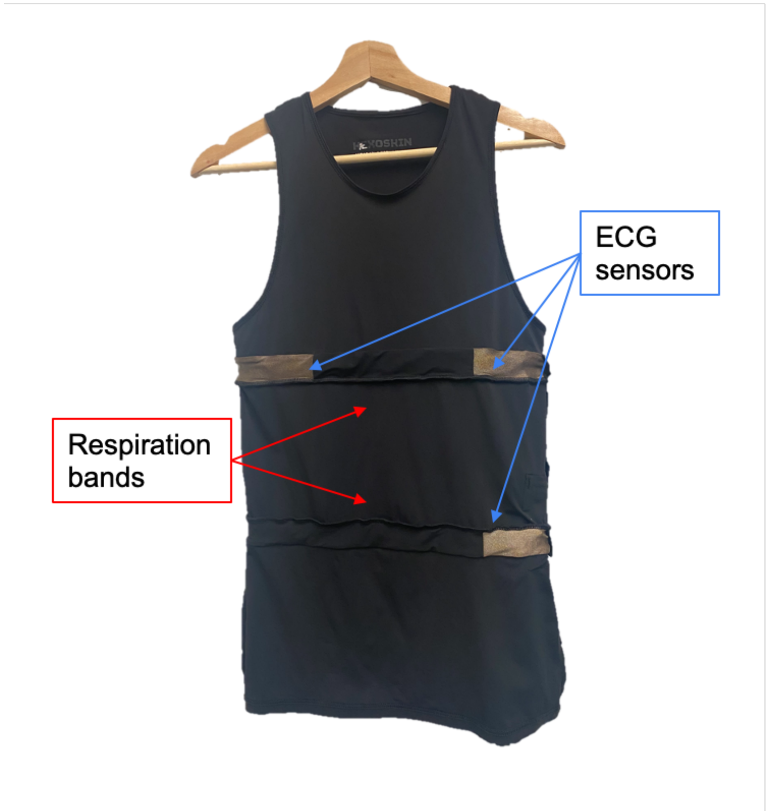

Supplementary Figure 1. Inverted Hexoskin smart shirt displayed with inner electrocardiogram (ECG) sensors and respiration bands visible.
